# Sexual Dimorphism in Vertebral Number: Developmental Implications Inferred From Vertebral and Body Proportions

**DOI:** 10.1111/ede.70062

**Published:** 2026-09-09

**Authors:** Kazunori Yamahira, V. K. Anoop, Rajeev Raghavan, Yoshitaka Tanaka, Satoshi Ansai

**Affiliations:** ^1^ Tropical Biosphere Research Center University of the Ryukyus Okinawa Japan; ^2^ Department of Fisheries Resource Management Kerala University of Fisheries and Ocean Studies Kochi India; ^3^ Ushimado Marine Institute Okayama University Okayama Japan

**Keywords:** axial patterning, developmental mechanisms, latitude, *Oryzias*, segmentation interval, vertebral length

## Abstract

Segmentation is a fundamental feature of vertebrate body plans, and variation in vertebral number contributes substantially to morphological diversity. Although sexual dimorphism in vertebral number has been documented in several vertebrate lineages, its developmental basis remains poorly understood. Here, we report pronounced sexual dimorphism in vertebral number in the western Indian endemic ricefish *Oryzias setnai*, distributed across ~1800 km. Radiographic analyses of individuals collected throughout its range revealed that vertebral number increased with latitude in both sexes, while males consistently possessed more vertebrae than females irrespective of geographic origin. Size‐adjusted analyses showed that this dimorphism is associated with sex differences in head size and position‐dependent variation in vertebral length. However, positional allometric analyses demonstrated that both the magnitude and direction of these sex differences vary markedly during growth. In particular, among smaller individuals, posterior vertebrae were relatively shorter in males than in females, suggesting reduced somite length in the posterior body axis of male embryos and potentially reflecting sex‐specific differences in the dynamics of somitogenesis. Given that this variation arises between males and females within a single species sharing an essentially identical genetic background, *O. setnai* provides a rare empirical system not only for linking early developmental processes to intraspecific sexual variation in vertebrate body plans but also as a powerful model to investigate the developmental basis of body plan variation across species.

## Summary

The developmental basis of sexual dimorphism in vertebral number remains poorly understood. We show pronounced dimorphism in a fish, linked to position‐dependent variation in vertebral length. This system provides a model for the developmental basis of body plan evolution.

## Graphical abstract

The developmental basis of sexual dimorphism in vertebral number remains poorly understood. We show pronounced dimorphism in *Oryzias setnai*, linked to position‐dependent variation in vertebral length, providing a model for linking somitogenesis dynamics to body plan evolution.

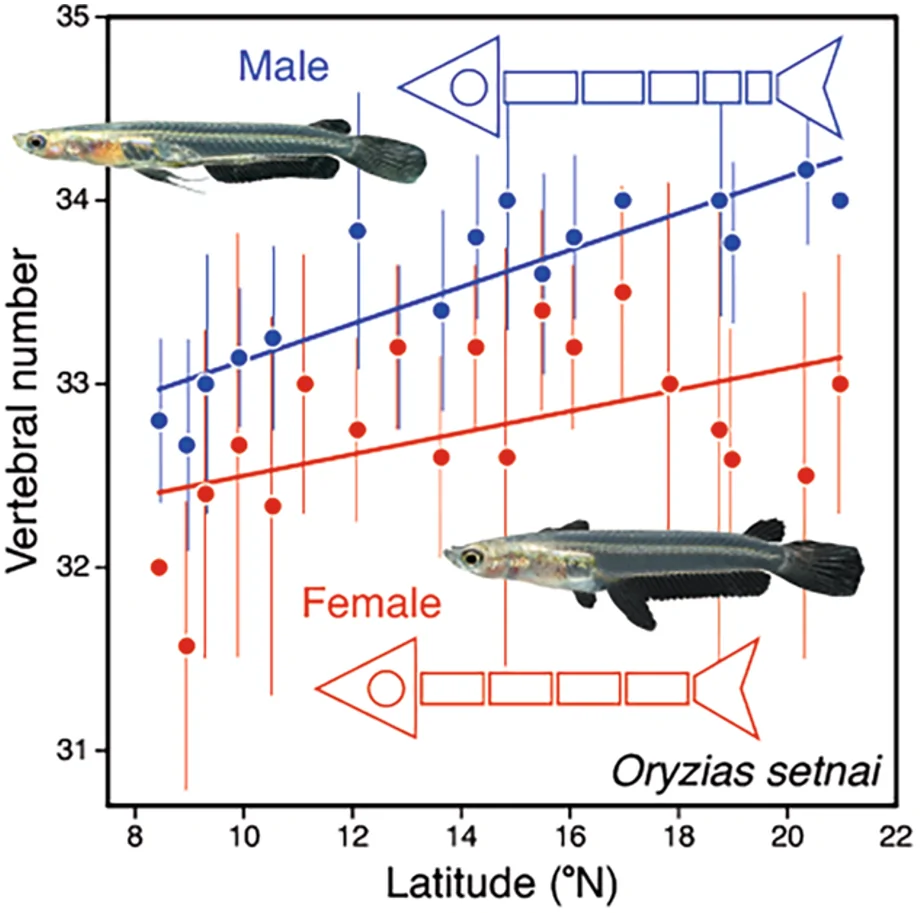

## 1 Introduction

Segmentation is a fundamental feature of animal body plans, contributing significantly to morphological diversity across taxa (Tautz 2004; Davis et al. 2007; Barresi and Gilbert 2023). In vertebrates, variation in the number of somites, the embryonic precursors to vertebrae, underlies much of their morphological disparity (Pourquié 2003; Gomez et al. 2008). Because somitogenesis is initiated and completed early in embryogenesis, prior to the differentiation of many organs including the gonads (e.g., Pourquié 2003), the evolution of sexual dimorphism in vertebral number is expected to be constrained. Nevertheless, sexual dimorphism in vertebral number has been documented in several vertebrate lineages, particularly snakes (Shine 2000) and some lizards (Porkert and Gosserova 1986; Kaliontzopoulou et al. 2008), demonstrating that sex‐specific developmental regulation can influence axial morphology.

To date, no instances of sexual dimorphism in vertebral number have been reported in mammals, birds, or turtles. In addition to snakes and some lizards, several examples have also been documented in urodeles and fishes (e.g., Lindsey 1975; Ficetola et al. 2013). Among fish, such dimorphism has been reported in eight species (Lindsey 1975; Castle and Böhlke 1976; Reimchen and Nelson 1987; Tsuji 2007), though the differences are typically minor, usually less than 2% on average (Lindsey 1975). A notable exception is the spaghetti eel (*Moringua edwardsi*), in which males and females differ by as much as 6%, and their vertebral count distributions show little to no overlap (Castle and Böhlke 1976). Nevertheless, relatively few studies have explicitly examined sexual dimorphism in vertebral number in fishes.

**Figure 1:**
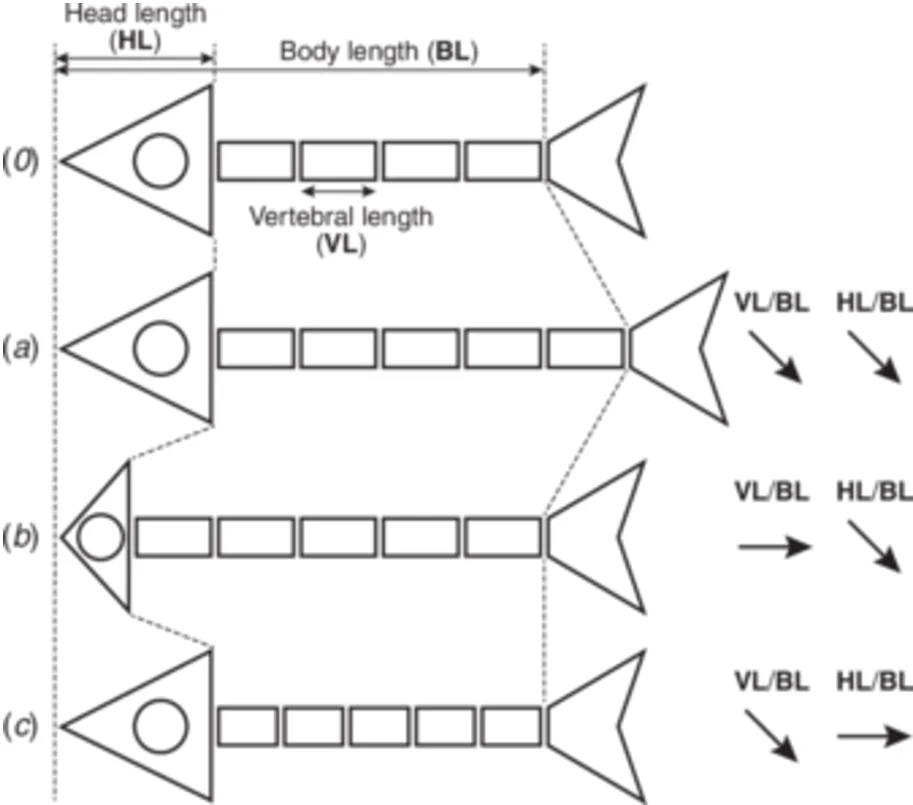
Conceptual diagram illustrating non‐mutually exclusive morphological components that may contribute to sexual dimorphism in vertebral number and the corresponding idealized patterns in relative vertebral length and relative head length. The three components shown are (a) sexual differences in the length of individual vertebrae, (b) sexual differences in head length, and (c) an increase or decrease in vertebral number independent of (a, b). For each component, the expected pattern is illustrated relative to a baseline condition (0) in which vertebral number differs by one between the sexes. The diagrams are intended as simplified representations for interpreting empirical patterns.

Sexual dimorphism in vertebral number is thought to involve variation in several non‐mutually exclusive morphological components, namely (*a*) sexual differences in axial length (the vertebral region), (*b*) sexual differences in head length, and (*c*) sexual differences in the length of individual vertebrae (Figure 1). Each of these components is expected to be expressed as a distinct idealized morphological pattern in relative vertebral length and relative head length with respect to body length (Figure 1). In addition, both the addition or deletion of vertebrae and sexual differences in the length of individual vertebrae may be localized to particular positions along the body axis. However, to our knowledge, no previous study has explicitly examined how observed sexual dimorphism in vertebral number arises by comparing relative vertebral lengths and body proportions.

Furthermore, vertebral length and body proportions may change in association with the development of secondary sexual characteristics. In contrast, vertebral number itself is thought to be determined during the period of somitogenesis (Gomez et al. 2008). Because somites are formed sequentially as the body axis elongates from anterior to posterior during embryogenesis, the sexual dimorphism in vertebral number is predicted to result from differences in the temporal dynamics of somitogenesis (i.e., differences in the rate of somite formation or in the timing of termination of somite production). Therefore, by clarifying how vertebral length and body proportions change during growth and by comparing these traits at juvenile stages around the onset of secondary sexual characteristics, it should be possible to test for developmental differences underlying the sexual dimorphism in vertebral number.

**Figure 2:**
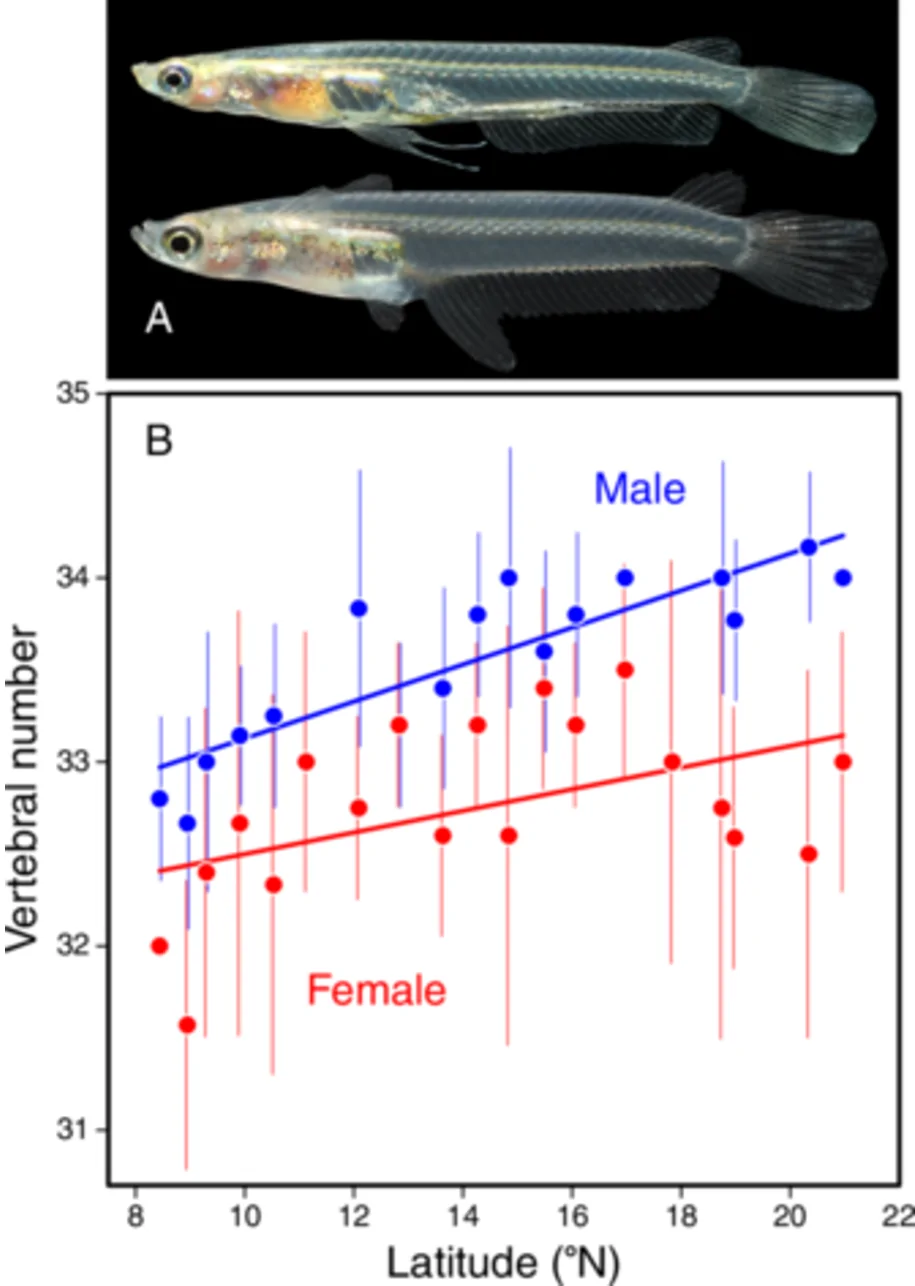
Sexual dimorphism in vertebral number in *Oryzias setnai*. (A) A male (top) and female (bottom) *Oryzias setnai*. (B) Relationship between latitude and the mean number of vertebrae among 19 populations of *Oryzias setnai* for males and females separately. Means were calculated from all individuals sampled in each population. Error bars indicate ±1 SD. Solid lines represent linear regression fits. [Color figure can be viewed at wileyonlinelibrary.com]

In this study, we report pronounced sexual dimorphism in vertebral number in the western Indian endemic ricefish *Oryzias setnai* (Figure 2a). This species exhibits a broad latitudinal distribution spanning approximately 1,800 km along the western coastal plains of India (Silas 1959; Jayaram 1981; Gopi et al. 2004; Parenti 2022; Anoop et al. 2025). In fishes with such wide latitudinal distributions, the potential influence of developmental temperature cannot be ignored when examining mechanisms underlying sexual differences in vertebral number, because lower temperatures during development generally tend to increase vertebral counts (Itazawa 1959). This relationship partly explains Jordan's rule (Jordan 1891), which states that populations at higher latitudes often exhibit more vertebrae (Lindsey 1988; McDowall 2008; Yamahira and Nishida 2009; Hara et al. 2026). Accordingly, we first examine both sexual dimorphism and latitudinal variation in vertebral number using specimens collected across the species' distributional range. We then conduct detailed analyses of sexual differences in individual vertebral lengths, body proportions, and their allometric relationships, to clarify how sexual dimorphism in vertebral number in adults is produced by differences in axial structure, and to infer the potential developmental mechanisms underlying these differences.

## 2 Materials and Methods

### 2.1 Field Collection

To cover most of the distribution range of *Oryzias setnai*, we collected specimens from 19 brackish‐water sites along the western coast of India (Supporting Information S1: Table S1). Scoop nets with a 1‐mm mesh size were used for sampling, and all collected individuals were fixed and preserved in 5% formalin. Sex was determined from external morphology, as males possess a modified anal fin that forms an intromittent organ (Kulkarni 1940; Parenti 2008).

### 2.2 Counting and Measuring Vertebrae

From each sampling site, five males and five females were randomly selected from the collected individuals for radiographic imaging. When fewer than 5 individuals of either sex were available, the deficit was compensated by including individuals of the opposite sex, resulting in a total of 10 individuals per site where possible; otherwise, all available individuals were examined. For the population from Mumbai (St. 04), the type locality of this species, 30 individuals (13 males and 17 females) were examined. In total, radiographic images were obtained from 200 individuals collected from 19 sites (Supp orting Information S1: Table S1) using a Softex E‐3 soft X‐ray system and industrial X‐ray film (Envelopak IX, Fujifilm).

Radiographic images were digitized and used to count the number of vertebrae in each individual; the urostyle was not counted as the terminal caudal vertebra. In addition, for each collection site, five individuals were randomly selected (adjusted whenever possible to include two or three males and two or three females) and the length of each vertebra was measured using Adobe Illustrator (version 30.1). For the population from Mumbai (St. 04), all collected individuals were exceptionally included in these measurements, and vertebral lengths were obtained for every individual. Head length, defined as the distance from the tip of the snout to the first vertebra, was also measured for all individuals included in the vertebral length analyses. The sum of vertebral lengths and head length was used as an index of overall body length. All vertebral and head‐length measurements were performed by a single observer.

### 2.3 Data Analysis

For males and females separately, we analyzed the relationship between the mean number of vertebrae at each collection site and latitude, as well as sexual differences in this relationship, using an analysis of covariance (ANCOVA) with latitude as a covariate. In addition, using all individuals for which vertebral length was measured, we tested for sexual differences in mean vertebral length (averaged across all vertebrae) and head length by ANCOVA with body length as a covariate.

Furthermore, to enable direct positional comparisons of homologous vertebrae among individuals, we focused on individuals with 33 vertebrae, the vertebral number with the largest combined sample size of both sexes (see Results), and examined which positions along the body axis exhibit sexual differences in vertebral length by calculating the mean relative length of each vertebra (scaled to body length) from anterior to posterior and comparing these values between sexes. We tested for sexual differences in the relative length of individual vertebrae and for sex‐specific changes along the body axis using a partially nested three‐way analysis of variance (ANOVA), with sex and vertebral position as fixed factors and individual nested within sex as a random factor.

We conducted separate ANCOVAs for selected anterior and posterior positions along the vertebral column. To reduce local variation arising from radiographic image quality and measurement error, vertebral lengths were averaged within non‐overlapping groups of three consecutive vertebrae, except for the first and terminal vertebrae, which were analyzed individually. Specifically, the anterior positions comprised the first vertebra, vertebrae 2–4, vertebrae 5–7, whereas the posterior positions comprised the terminal vertebra, the second to fourth vertebrae from the posterior end, and the fifth to seventh vertebrae from the posterior end. Vertebral length (or the mean length of each vertebra group) was used as the dependent variable, body length as a covariate, and sex as a fixed factor, including the sex × body length interaction.

All statistical analyses were conducted using JMP version 13.2.0 (SAS Institute Inc., Cary, NC, USA).

## 3 Results

In both males and females, the number of vertebrae increased in populations at higher latitudes; however, at all latitudes except one, males consistently had a higher mean vertebral count than females (Figure 2). An analysis of covariance (ANCOVA) with latitude as a covariate revealed a statistically significant effect of latitude on vertebral number (*F*_1,32_ = 27.7087, *p* < 0.0001), as well as a significant sexual difference in vertebral number (*F*_1,32_ = 46.4714, *p* < 0.0001). In contrast, the interaction between sex and latitude was not significant (*F*_1,32_ = 1.9068, *p* = 0.1769), indicating that the slope of the relationship between latitude and mean vertebral number did not differ between the sexes.

**Figure 3:**
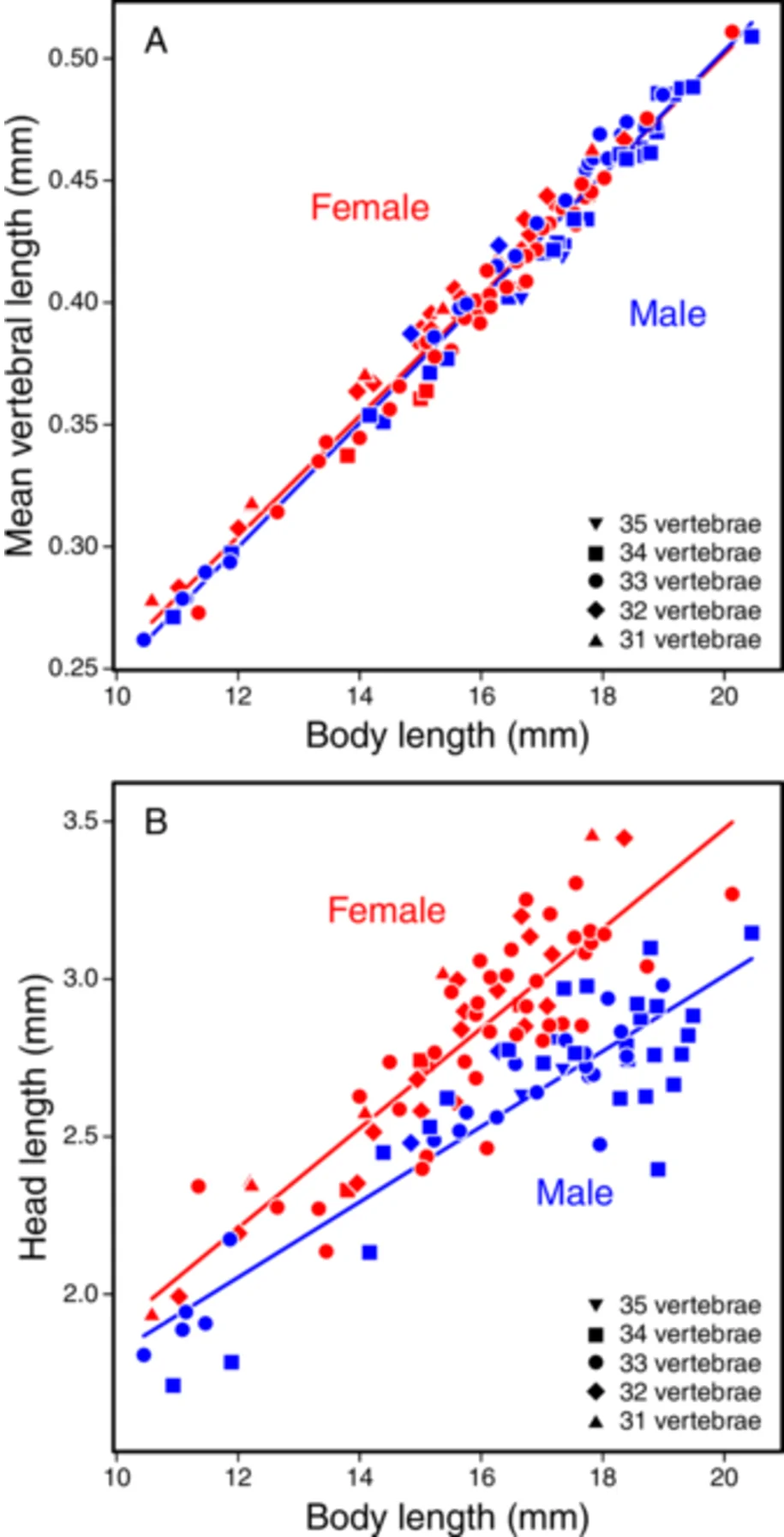
Relationships between body length and vertebral length, and head length in *Oryzias setnai*, shown separately for males and females. (A) Mean vertebral length (averaged across all vertebrae) versus body length. (B) Head length versus body length. Different symbols denote individuals with different vertebral numbers. Solid lines represent linear regression fits. [Color figure can be viewed at wileyonlinelibrary.com]

Across all populations, there was no significant sexual difference in mean vertebral length relative to body length, as indicated by a nonsignificant ANCOVA (*F*_1,117_ = 0.6078, *p* = 0.4372) (Figure 3a). By contrast, head length relative to body length was significantly smaller in males than in females (ANCOVA, *F*_1,117_ = 114.9081, *p* < 0.0001) (Figure 3b). However, the slope of the relationship between head length and body length differed significantly between the sexes (*F*_1,117_ = 8.4300, *p* = 0.0044), with the sexual difference in head length diminishing in smaller individuals.

**Figure 4:**
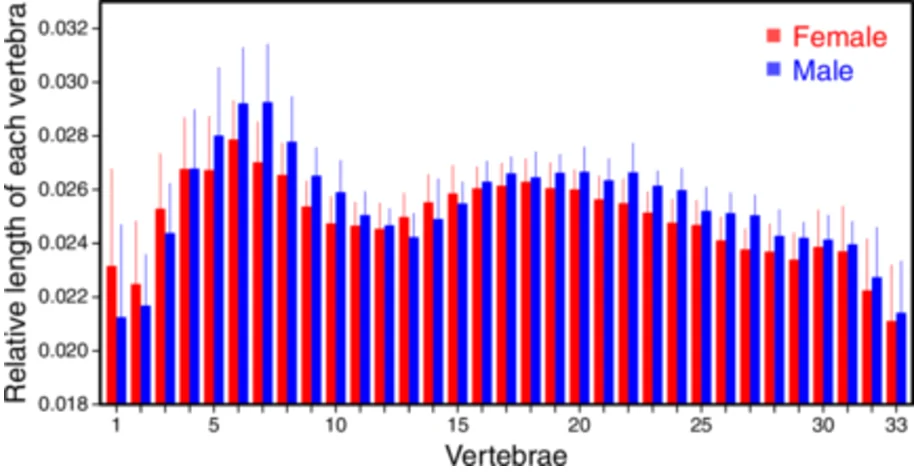
Mean relative length of each vertebra (scaled to body length) along the anteroposterior axis in *Oryzias setnai* individuals with 33 vertebrae, shown separately for males and females. Error bars indicate ±1 SD. [Color figure can be viewed at wileyonlinelibrary.com]

The range of vertebral number was 32–35 in males and 31–34 in females, and the vertebral count with the largest combined sample size of males and females was 33. Focusing on individuals with 33 vertebrae, we found that both the direction and magnitude of sexual differences in vertebral length varied markedly along the body axis (Figure 4). In the anterior region of the body axis (vertebrae 1–4), individual vertebrae were shorter in males than in females, with the difference being most pronounced in the first vertebra. In the subsequent mid‐anterior region (vertebrae 5–12), vertebrae were longer in males than in females, whereas in three mid‐body vertebrae (vertebrae 13–15) females had slightly longer vertebrae. From this point posteriorly (vertebrae 16–33), vertebrae were consistently longer in males than in females. The partially nested three‐way ANOVA revealed a significant main effect of vertebral position (*F*_32,1824_ = 75.0570, *p* < 0.0001) but no significant main effect of sex on relative vertebral length (*F*_1,57_ = 0.0007, *p* = 0.9791). However, the sex × vertebral position interaction was significant (*F*_32,1824_ = 4.8703, *p* < 0.0001), indicating that sexual differences in vertebral length vary along the body axis.

**Figure 5:**
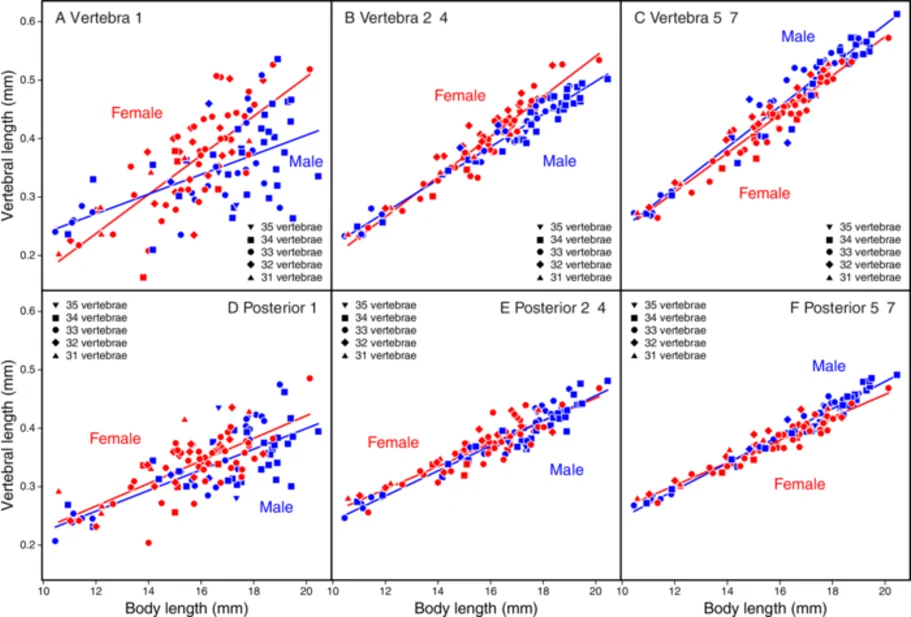
Relationships between body length and vertebral length at different positions along the body axis in *Oryzias setnai*, shown separately for males and females. (A) Length of the first vertebra. (B) Mean vertebral length of vertebrae 2–4. (C) Mean vertebral length of vertebrae 5–7. (D) Length of the terminal vertebra. (E) Mean vertebral length of the second to fourth vertebrae counted from the posterior end. (F) Mean vertebral length of the fifth to seventh vertebrae from the posterior end. Different symbols denote individuals with different vertebral numbers. Solid lines represent linear regression fits. [Color figure can be viewed at wileyonlinelibrary.com]

The allometric relationship between individual vertebral length and body size varied markedly along the body axis. In the first vertebra, larger individuals had longer vertebrae in females than in males, whereas in smaller individuals, males had longer vertebrae than females (Figure 5a). An ANCOVA revealed that this size‐dependent reversal of sexual dimorphism was statistically significant (*F*_1,117_ = 11.7334, *p* < 0.0001). A similar pattern was observed in the other anterior vertebrae (vertebrae 2–4) (Figure 5b; ANCOVA, *F*_1,117_ = 17.8729, *p* < 0.0001). However, this pattern disappeared in vertebrae 5–7, where males had significantly longer vertebrae than females regardless of body size (Figure 5c; ANCOVA, *F*_1,117_ = 14.4099, *p* < 0.0001). In contrast, in the terminal vertebra, males had shorter vertebrae than females across all body sizes, and this sexual difference was statistically significant (Figure 5d; ANCOVA, *F*_1,117_ = 4.7116, *p* = 0.0320). Similarly, in the second to fourth vertebrae counted from the posterior end, males tended to have shorter vertebrae than females, particularly in smaller individuals (Figure 5e), although the difference was not statistically significant (ANCOVA, *F*_1,117_ = 1.0803, *p* = 0.3008). In the fifth to seventh vertebrae from the posterior end, the size‐dependent reversal of sexual dimorphism (males > females in larger individuals; males < females in smaller individuals) became pronounced (Figure 5e), and this reversal was statistically significant (ANCOVA, *F*_1,117_ = 15.8107, *p* = 0.0001).

## 4 Discussion

### 4.1 Geographic Patterns and the Genetic Basis of Sexual Dimorphism

For both males and females, the number of vertebrae was higher in northern populations, consistent with Jordan's rule (Jordan 1891). At all locations, however, males had more vertebrae than females. Although vertebral number in fishes is often influenced by developmental temperature (Itazawa 1959), the latitudinal range sampled in this study falls within a tropical monsoon climate, where annual temperature variation is limited and mean temperatures are relatively uniform across the study sites (Peel et al. 2007). For example, the annual mean temperatures at Mumbai and Kochi, two localities separated by approximately 1000 km, are nearly identical (27.9°C and 27.8°C, respectively; National Centers for Environmental Information 2023). This suggests that the observed sexual dimorphism in vertebral number is consistent with a genetic basis, although confirmation will require common‐garden experiments.

Vertebral number is known to influence body flexibility and swimming performance in fishes (e.g., Brainerd and Patek 1998; McDowall 2003). Thus, the observed sexual differences in vertebral number, as well as the latitudinal variation documented in this study, may have functional consequences for swimming performance. Although these possibilities remain speculative, they provide testable hypotheses for future studies.

### 4.2 Axial Position‐Dependent Variation in Vertebral Length and Developmental Implications

Focusing on individual vertebral lengths and body proportions, no significant sexual difference was detected in mean vertebral length relative to body length, whereas relative head length was significantly smaller in males than in females. These results indicate that the observed sexual dimorphism in vertebral number is primarily attributable to component (*b*) in Figure 1, namely sexual differences in head length. However, because the sexual difference in head length disappeared among smaller individuals, this difference is also shown to emerge during growth and the development of secondary sexual characteristics. Moreover, sexual differences in individual vertebral length varied in both direction and magnitude along the body axis. Together, these findings suggest that examining how individual vertebral lengths change during growth, at each position along the body axis, provides a means to test developmental differences underlying sexual dimorphism in vertebral number.

Interestingly, sexual differences in the allometric relationships between individual vertebral length and body size differed markedly depending on axial position. In the most anterior region of the body axis (vertebrae 1–4), vertebrae were shorter in males than in females among larger individuals, whereas this sexual difference was reversed among smaller individuals, with males having longer vertebrae. In the subsequent vertebrae (vertebrae 5–7), males consistently had longer vertebrae than females regardless of body size. By contrast, in the posterior region (the first to fourth vertebrae counted from the posterior end), males consistently exhibited shorter vertebrae than females irrespective of body size. In the vertebrae located immediately anterior to this region (the fifth to seventh vertebrae counted from the posterior end), sexual differences changed with body size, such that males had longer vertebrae among larger individuals but shorter vertebrae among smaller individuals. These results indicate that, among smaller individuals, vertebrae in the anterior portion of the body axis are longer in males than in females, whereas vertebrae in the posterior portion are shorter in males. Across the vertebral column as a whole, vertebrae that are shorter in males may predominate, potentially resulting in the observed sexual difference in vertebral number.

Vertebral number and size are largely determined by the properties of somites (Brainerd and Patek 1998; Cooke and Zeeman 1976). Therefore, the higher vertebral number in males implies that male embryos produce more somites than female embryos. This could result from increasing the number of segmentation clock cycles completed before somitogenesis ends, either by shortening the clock period, prolonging the duration of somitogenesis, or both. Because the duration of somitogenesis depends on the continued presence of posterior axial progenitors, the latter mechanism could result from slower depletion or prolonged maintenance of this progenitor population in males, thereby extending segmentation and permitting additional somites to form. In other vertebrates, the maintenance and depletion of posterior progenitors are regulated in part by the Gdf11–Lin28–Hox13 network (Aires et al. 2019; Robinton et al. 2019), making this pathway a plausible candidate for mediating sex‐specific differences in somite number. Distinguishing a clock‐period‐based mechanism from a progenitor‐persistence‐based mechanism will require direct comparison of segmentation‐clock dynamics and posterior progenitor maintenance between male and female embryos, for example, through live imaging of clock reporters together with time‐resolved measurements of axial progenitor abundance, presomitic mesoderm dynamics, and the timing of somitogenesis termination.

### 4.3 Early Axial Patterning, Sex‐Determination Systems, and Evolutionary Implications

The presence of sexual dimorphism in vertebral length around the onset of secondary sexual differentiation is consistent with the prediction that its origin lies in sex differences in early axial patterning during embryogenesis. Vertebrate somitogenesis is known to be completed prior to the differentiation of many other organs, including the gonads (e.g., Pourquié 2003). Therefore, the sex‐specific differences influencing early axial patterning are unlikely to be secondarily induced by sex hormones and are more likely specified at earlier developmental stages. One possibility is that sex‐determining genes exert pleiotropic effects on early axial patterning or somitogenesis. For example, in the medaka *Oryzias latipes*, the sex‐determining gene *dmy* has been reported to show low‐level expression outside the developing gonads (Ohmuro‐Matsuyama et al. 2003), suggesting that the sex‐determining locus may influence embryonic processes beyond gonadal differentiation. Alternatively, linkage disequilibrium between the sex‐determining locus and genes involved in axial patterning or somitogenesis may generate consistent sexual differences in vertebral number. Given the diversity of sex‐determining systems among medaka species (Myosho et al. 2015; Ansai et al. 2022), identifying the sex‐determining gene in *Oryzias setnai* will be essential for evaluating these possibilities. This hypothesis is also consistent with the absence of sexual dimorphism in vertebral number in mammals and birds, where sex‐determination systems are highly conserved and sex chromosomes are stable (Smith et al. 1999; Wallis et al. 2008; Graves 2014).

Although we recognize that explicitly distinguishing abdominal and caudal vertebrae would be beneficial, given their differing developmental patterns and functional roles (e.g., Yamahira et al. 2009), this is difficult in *O. setnai*, because males possess a modified anal fin that forms an intromittent organ (Kulkarni 1940; Parenti 2008), with articulations extending deeply into the body cavity. These morphological features make it difficult to apply identical anatomical criteria for delimiting abdominal and caudal vertebrae between sexes, but they also provide a unique opportunity to examine how sex‐specific functional demands interact with early axial patterning during development. Importantly, because this variation arises between males and females of a single species that share an essentially identical genetic background, *O. setnai* offers a rare opportunity to investigate the developmental mechanisms generating body plan variation without the confounding effects of extensive genetic divergence typically associated with interspecific comparisons. In this way, *O. setnai* provides a rare empirical system not only for linking early developmental processes to intraspecific sexual variation in vertebrate body plans but also as a powerful model to investigate the developmental basis of body plan variation across species.

## Ethics Statement

This study was conducted following the Regulation for Animal Experiments at the Kerala University of Fisheries and Ocean Studies (KUFOS), Kochi, India, and with permission from the National Biodiversity Authority (NBA), Government of India (permission number: INBAB202204012/22/22‐23/2649).

## Conflicts of Interest

The authors declare no conflicts of interests.

## Supporting information


**Table S1:** List of individuals and raw vertebral count and measurement data for *Oryzias setnai* used in this study.

## Data Availability

All raw data are provided in Supporting Information S1: Table S1.
